# Incidence, outcomes, and predictors of new heart failure in syrian conflict-affected population following hospitalization for atrial fibrillation: A retrospective cohort study

**DOI:** 10.1177/02676591241283883

**Published:** 2024-09-10

**Authors:** Ibrahim Antoun, Alkassem Alkhayer, Majed Aljabal, Yaman Mahfoud, Alamer Alkhayer, Ahmed I Kotb, Joseph Barker, Riyaz Somani, G André Ng, Mustafa Zakkar

**Affiliations:** 1 Department of Cardiovascular Sciences, 150459 University of Leicester, Leicester, UK; 2 Faculty of Medicine, University of Aleppo, Aleppo, Syria; 3 594809 University of Tishreen’s Hospital, Latakia, Syria; 4 9632 Leicestershire Partnership NHS Trust, Leicester, UK; 5 National Heart and Lung Institute, 90897 Imperial College London, London, UK; 6 Department of Cardiac Surgery, University Hospitals of Leicester NHS Trust, Glenfield Hospital, Leicester, UK; 7 573774 NIHR Leicester Biomedical Research Centre, Leicester, UK; 8 Department of Cardiology, University Hospitals of Leicester NHS Trust, Glenfield Hospital, Leicester, UK; 9 Faculty of Medicine, University of Damascus, Damascus, Syria

**Keywords:** atrial fibrillation, mortality, readmission, heart failure, Syria, conflict

## Abstract

**Background:**

Atrial fibrillation (AF) is the most common arrhythmia worldwide. Data regarding readmission for new congestive heart failure (CHF) following index admission for AF in the developing world are poorly described.

**Objectives:**

The study aimed to assess the rate, predictors, and outcomes of 120-day CHF readmission after index admission for AF in Syria.

**Methods:**

This retrospective cohort study collected all adult patients without known CHF who had an index admission with AF to Latakia’s tertiary center between June 2021-December 2023. Data were taken from patients’ medical notes. The primary outcome included readmission with incident CHF within 120 of index discharge, and secondary outcomes included predictors and outcomes of these CHF readmissions.

**Results:**

A total of 660 patients were included in the final analysis, of which 69 (11.7%) were readmitted with new CHF within 120 days of index discharge. Readmitted patients had higher median age (58 vs 70 years, *p* < .001). Factors that independently increased 120-day CHF incidence were age ≥60 years (HR: 9.8, 95% CI: 4.8-23.6, *p* < .001), diabetes mellitus (DM) (HR:2.9, 95% CI:1.7-4.9, *p* < .001), valvular heart disease (VHD) (HR:1.7, 95% CI:1.04-2.78, *p* = .047), and hypertension (HR:2.5, 95% CI:1.5-4, *p* < .001). Inpatient mortality occurred in six readmitted patients (9%). LVEF <40% (HR:6.7, 95% CI: 24.31, *p* = .01) and DM (HR:7.2, 95% CI: 1.9-33, *p* = .004) were independently associated with inpatient mortality.

**Conclusion:**

Hospitalization for new CHF was common in Syrian patients discharged with AF. The clinical predictors of incident CHF emphasize the importance of integrated management of lifestyle risk factors and common comorbidities in AF patients to optimize outcomes in resource-depleted communities.

## Key findings

• The 120-day readmission rate with incident heart failure after index admission for atrial fibrillation in this Syrian center was 11.7%.
• Older age, valvular heart disease, diabetes, and hypertension increase the risk of incident heart failure.
• Inpatient mortality with heart failure was 9% with diabetes and impaired systolic function being predictive of mortality.

## Introduction

Atrial fibrillation (AF) is the most common sustained arrhythmia worldwide, and its prevalence in low to middle-income countries is likely underestimated.^1^ There is little data on AF demographics and management in the Middle East, with only four epidemiological data registries.^2^ Furthermore, AF-related research in the Arab world contributed only 0.7% of the total AF research.^3^ Syria has been embroiled in conflict since 2011. The country is undergoing a humanitarian crisis with mass displacement of large portions of the population while the conflict continues. It has been deprived of healthcare resources and funding, particularly exacerbated during the COVID-19 and cholera outbreaks.^4,5^ As a result, less than half of its hospitals operate at usual performance, with over 50% of its healthcare workforce forced to leave.^6^ AF management in hospitals during the current political and economic turmoil is unclear, with only one recently published inpatient outcomes and figures originating from Syrian hospitals demonstrating a relatively younger inpatient population with an inpatient mortality of 4.7 %.^7^ In the context of the resource constraints, a real-world depiction of the current AF care and observed outcomes can aid the management and allocation of resources. This can be achieved by identifying remediable deficiencies and, more importantly, reasonable and practical solutions that could be implemented. CHF and AF share underlying pathophysiological mechanisms and risk factors and often coexist. Former studies have reported that the epidemiological relationship between CHF and AF is bidirectional, meaning that prevalent AF has a high risk of incident CHF and vice versa.^8^ Proposed mechanisms of CHF in AF patients include decreased density of β-adrenergic receptors and L-type calcium channels, increased diastolic contracture, and increased left ventricular diastolic pressure, causing impaired myocardial blood flow.^9^

Furthermore, the incidence of CHF in AF patients is associated with adverse outcomes.^10,11^ The incidence and predictors of incident CHF after AF admissions have been studied in the developed world but not in a developing country under conflict circumstances.^10^ This study aims to assess the incidence of CHF within 120 days of index admission with AF to Latakia’s tertiary care center in Syria.

## Methods

This is a single-centre retrospective observational cohort study conducted at Tishreen’s University Hospital, Latakia, Syria, between the 1^st^ of June 2021 and the 1^st^ of December 2023. The study included patients over 18 years old treated with AF as the primary diagnosis in the indexed admission. Patients with known CHF were excluded. Patients under 18 years and with missing data for age and sex were excluded. Patients are followed for 120 days following discharge from their index admission for readmission with incident CHF. Data sources include hospital paper and electronic records. Index admission and readmission causes were determined by the medical consultant or the medical registrar after discussing the case verbally with the medical consultant.

The primary outcome of our study was incident CHF readmissions within 120 days. A secondary analysis explored inpatient mortality and predictors of 120‐day readmission with new CHF and inpatient mortality. Readmission causes were identified using the impression of the medical registrar after a discussion with the medical consultant. The CHF diagnosis was based on clinical signs and symptoms of congestion, such as dyspnea, rales on lung auscultation, fluid retention, jugular venous distention, and other radiographic features of pulmonary edema. Among patients with incident CHF, left ventricular ejection fraction (LVEF) reported using transthoracic echocardiogram (TTE) on the admission of incident CHF diagnosis was used to identify CHF subtypes: heart failure with preserved ejection fraction (HFpEF) (EF ≥50%) and heart failure with reduced ejection fraction (HFrEF) (EF <50%). In AF patients, the TTE was conducted when the heart rate was <110 beats per minute to avoid false low LVEF. Due to financial constraints, B-type natriuretic peptide was not performed in the hospital during the study period. The research reported in this article adhered to the Declaration of Helsinki. The project was conducted as a part of an audit approved by the hospital board and involved prospective analysis of retrospectively collected anonymized data (reference: 305/A). Therefore, the need for consent was waived by the hospital board.

### Statistical analysis

Continuous variables are expressed as median and interquartile ranges (IQR). Categorical variables are expressed as counts and percentages (%). Pearson’s χ 2 or Fisher’s exact test was used for categorical variables between groups. Students’ t-tests and Kruskal-Wallis tests were used to compare continuous variables between the groups depending on the normality of the distribution.

Cox regression and Kaplan-Meier models investigated the relationship between new CHF readmissions, CHF inpatient mortality, and variables. We hypothesized that specific demographic characteristics and comorbidities would affect 120-day CHF readmission probability and inpatient mortality with CHF. Therefore, a base model was constructed consisting of age and gender to assess the incremental value of comorbidities that are significantly associated with 120-day incident CHF readmission and inpatient mortality. Statistically significant comorbidities in the univariate analysis were added to the base model in multivariable analysis to improve the predictability. A 2-sided *p*-value <.05 was considered statistically significant. Statistical analysis was performed using GraphPad Prism V10.0 for Mac (San Diego, California, USA).

## Results

### Patient characteristics

Our study included 660 consecutive patients with an index admission with AF as the primary diagnosis between June 2021 and December 2023 without a prior history of CHF. Among these, 69 patients (11.7%) were readmitted with incident CHF within 120 days of index primary AF discharge. Demographics stratified by CHF readmission are demonstrated in Table 1. Compared to patients without CHF readmission, the CHF patients were older (median age 70 vs median age 58 years, *p* < .001), were more likely to have diabetes mellitus (DM) (38% vs 19%, *p* = .001), and valvular heart disease (VHD) (25% vs 16%, *p* = .004). 62 (90%) were in AF on readmission in the CHF arm. Only readmitted CHF patients had TTE done during readmission. They demonstrated an LVEF of ≥50% in 7 patients (10%) and diagnosed with HFpEF, while the rest had an LVEF of <50%, of which half had LVEF< 40%.

**Table 1.** Demographics of study subjects stratified by the first heart failure hospitalization within 120 days after index discharge for atrial fibrillation.

	Overall (*n* = 660)	CHF admission (*n* = 69)	No CHF readmission (*n* = 591)	*p*-value
Demographics, *n* (%) or median (IQR)				
Age (years)	60 (53-67)	70 (66-78)	58 (52-65)	<0.001
Male	361 (55%)	39 (57%)	322 (54%)	0.78
Cardiovascular comorbidities, *n* (%)				
Hypertension	199 (30%)	34 (49%)	165 (28%)	0.001
Ischemic heart disease	124 (19%)	13 (19%)	111 (19%)	0.99
Diabetes mellitus	140 (21%)	26 (38%)	114 (19%)	0.001
Cerebrovascular disease	85 (13%)	7 (10%)	78 (13%)	0.57
PCI Within the last year	32 (5%)	3 (4%)	29 (5%)	0.99
CABG within the last year	20 (3%)	3 (4%)	17 (3%)	0.45
Thyroid disease	49 (7%)	5 (7%)	44 (7%)	0.99
Valvular heart disease	113 (17%)	17 (25%)	96 (16%)	0.004
Other comorbidities, *n* (%)				
Anemia	101 (15%)	10 (14%)	91 (15%)	0.99
Dementia	44 (7%)	6 (9%)	38 (5%)	0.45
Active malignancy	25 (4%)	1 (1%)	24 (4%)	0.64
Chronic liver failure	56 (8%)	7 (10%)	49 (8%)	0.32
Chronic lung disease	75 (11%)	5 (7%)	70 (12%)	0.65
Chronic kidney failure	61 (9%)	4 (6%)	57 (10%)	0.38

CHF: congestive cardiac failure. IQR: interquartile range. PCI: primary coronary intervention. CABG: coronary artery bypass graft.

### Predictors of CHF incidence

**Figure 1.**
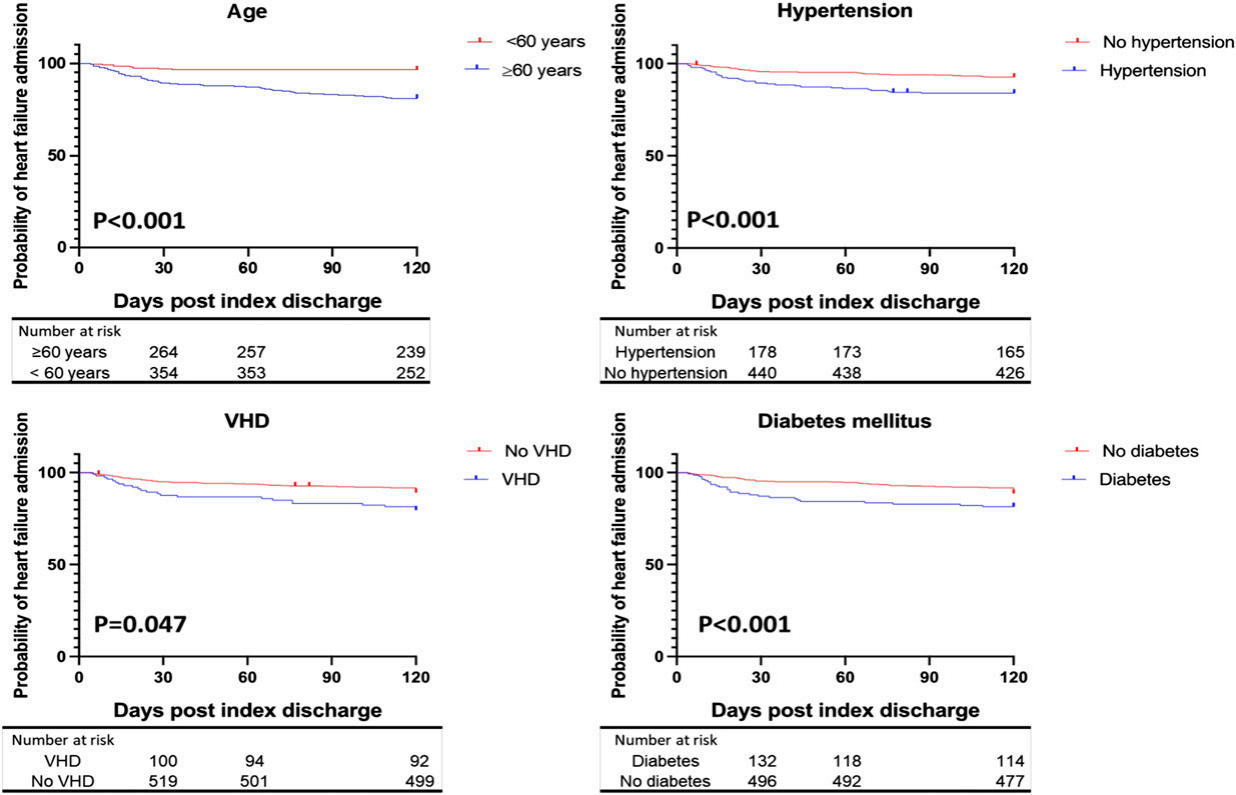
Demonstrates Kaplan-Meier analysis regarding positive predictors of first heart failure hospitalization within 120 days after index discharge for atrial fibrillation. VHD: valvular heart disease.

Regarding CHF incidence predictors, univariable Cox regression showed age ≥60 years (HR: 4.7, 95% CI: 2.9-7.7, *p* < .001), DM (HR: 1.96, 95% CI: 1.2-3.5, *p* = .01), hypertension (HR: 2.6, 95% CI: 1.5-2.8, *p* < .001), and VHD (HR: 2.4, 95% CI: 1.3-4.3, *p* = .003) were associated with increased probability of 120-days CHF readmission. Similarly, multivariate analysis showed that age ≥60 (HR: 9.8, 95% CI: 4.8-23.6, *p* < .001), DM (HR: 2.9, 95% CI: 1.7-4.9, *p* < .001), VHD (HR: 1.7, 95% CI: 1.04-2.78, *p* = .047), and hypertension (HR: 2.5, 95% CI: 1.5-4, *p* < .001) were independently associated with increased risk of 120-day CHF readmission, as demonstrated in Table 2. Kaplan-Meier analysis shown in Figure 1 revealed that We identified that age ≥60 years was associated with an increased risk for CHF readmission compared to the age of <60 years 120 days (19% vs 3.5%, *p* = 0 < 001). Similarly, the presence of VHD was associated with an increased risk of admission at 120 days (20.4% vs 10.3%, *p* = .047), DM at 120 days (18.6% vs 11.4%, *p* < .001), and hypertension at 120 days (16% vs 7.4%, *p* < .001).

**Table 2.** Cox regression showing univariate and multivariable-adjusted predictors of first hospitalization with heart failure within 120 days after index discharge for atrial fibrillation hospitalization.

CHF readmission within 120 days	Univariable analysis		Multivariable- adjusted	
Presenting characteristic	HR (95% CI)	*p*-value	HR (95% CI)	*p*-value
Female versus male	0.88 (0.67-1.2)	0.76	0.74 (0.45-1.24)	0.26
Age of or above 60 years versus age below 60 years	4.7 (2.9-7.7)	<0.001	9.8 (4.8-23.6)	<0.001
Hypertension (yes versus no)	2.6 (1.5-4.1)	<0.001	2.5 (1.5-4)	<0.001
Diabetes mellitus (yes versus no)	1.96 (1.2-3.5)	<0.001	2.9 (1.7-4.9)	<0.001
Valvular heart disease (yes versus no)	2.4 (1.3-4.3)	0.003	1.7 (1.04-2.78)	0.047
Cerebrovascular disease (yes versus no)	0.7 (0.35-1.3)	0.3		
Ischemic heart disease (yes versus no)	1.1 (0.56-1.95)	0.49		
Active thyroid disease (yes versus no)	0.79 (0.35-1.29)	0.23		
Chronic kidney disease (yes versus no)	1.15 (0.55-2.4)	0.79		
Chronic liver disease (yes versus no)	1.1 (0.47-2.3)	0.54		
Active malignancy (yes versus no)	0.6 (0.14-2.7)	0.23		
Chronic lung disease (yes versus no)	0.92 (0.47-1.8)	0.78		
Dementia (yes versus no)	0.9 (0.38-2.1)	0.76		
Anaemia (yes versus no)	0.74 (0.43-1.26)	0.28		

CI: confidence interval. HR: hazard ratio.

### Inpatient mortality of CHF and its predictors

Of the 69 patients readmitted with CHF, six patients (9%), all of which had HFrEF with LVEF <40%, died after a median of 8 days (4.8-13) due to cardiogenic shock and multiorgan failure. The rest were discharged after a median of 5 days^3–6^ of inpatient active treatment. We identified that LVEF <40% years was associated with an increased risk for inpatient mortality with CHF compared to LVEF ≥40% at day 12 of readmission of admission (0% vs 78%, *p* = .041). Similarly, the presence of DM was associated with an increased risk of inpatient mortality at day 12 readmission (12.5% vs 69%, *p* = .01). Regression analysis regarding inpatient mortality predictors is demonstrated in Table 3. Univariable Cox regression demonstrated LVEF <40% (HR: 5.5, 95% CI: 1.26-24, *p* = .041) and DM (HR: 6.5, 95% CI: 1.3-34, *p* = .01) as predictive of inpatient mortality as demonstrated. Similarly, multivariate analysis showed that LVEF <40% (HR: 6.7, 95% CI: 24.31, *p* = .01) and DM (HR: 7.2, 95% CI: 1.9-33, *p* = .004) were independently associated with inpatient mortality.

## Discussion

This is the first study describing trends and predictors of 120-day readmission with incident CHF following index admission with primary AF in Syria and the Middle East. This study highlights multiple significant novel findings for the Syrian population. First, more than one in 10 patients were readmitted with incident CHF after index discharge for primary AF. Second, age ≥60 years (HR: 9.8, 95% CI: 4.8-23.6, *p* < .001), DM (HR: 2.9, 95% CI: 1.7-4.9, *p* < .001), VHD (HR: 1.7, 95% CI: 1.04-2.78, *p* = .047), and hypertension (HR: 2.5, 95% CI: 1.5-4, *p* < .001) were independently associated with an increased risk of first CHF admissions. Third, inpatient mortality occurred in 9% of CHF readmissions, with LVEF <40% (HR: 6.7, 95% CI: 24.31, *p* = .01) and DM (HR: 7.2, 95% CI: 1.9-33, *p* = .004) were independently associated with inpatient mortality. As AF is known to have enormous implications on economies worldwide,^12^ recent studies have focused on many aspects of AF, including treatment patterns, hospitalization, and readmission rates.^13^ Our previous work on AF in Syria demonstrated that CHF was the second most common cardiac cause of readmission after AF recurrence.^14^ This cohort reached a cumulative new CHF readmission rate similar to that in Australia within 120 days only.^10^ Other studies from the United States did not exclude known CHF patients from the analysis. Therefore, a comparison could not be made.^15^ There are no data from the developing world to compare with. The higher CHF incidence rate in this study may be explained by the conflict in Syria since 2011, which has significantly diminished health infrastructure and displaced healthcare workers.^16^ Furthermore, primary care is severely impaired nationwide, and healthcare costs are high relative to income, which leads to medication non-compliance.^17^ Therefore, care is sought at the last minute, and comorbidities are poorly managed. Hospitals are understaffed with junior staff, and escalation of care within hospitals is limited with few high-dependency beds. As only 50% of hospital and primary health centers are fully functional in the country,^16^ managing risk factors and following up patients who presented to hospitals with acute AF after discharge is difficult. Furthermore, medication access has been challenging due to conflict and poverty.^18^ Engagement in high-risk behaviors, such as smoking, is well correlated with CHF incidence.^19^ A recent Syrian study during the conflict showed a smoking rate of 38% in 978 participants with a mean age of 25 years, which was described as worrying in the survey.^20^ Therefore, patient education and addressing these risk factors is vital to optimize outcomes and reduce CHF incidence in resource-depleted areas such as Syria. Although no data existed before the conflict, supporting the Syrian health care system, especially primary care, would help reduce the CHF incidence rate in Latakia and nationwide.

VHD, hypertension, older age, and DM were independent risk factors for incident CHF admission after index AF admission, mainly in keeping with the Australian data.^10^ This is not surprising, as all these factors are known to cause CHF in the literature.^21–23^ Therefore, managing these risk factors early could be vital in minimizing this relatively high CHF incidence.

Our inpatient mortality of 9% was more than double the mortality rate of CHF patients with AF in the United States registry of 3.6%^24^ without developing world inpatient data to compare to. The outcome difference is due to factors related to healthcare infrastructure, socioeconomic conditions, and ongoing conflict in Syria. DM and LVEF <40% were independent predictors of inpatient mortality regarding inpatients readmitted with CHF. The literature demonstrates the interaction of impaired LVEF and DM in CHF patients, which significantly amplifies the harmful effects of each as distinct disease entities.^25^ Our study is in keeping with data from the developed world showing a positive correlation between DM is associated with and inpatient mortality in CHF patients, particularly in HFrEF.^26^

These results should encourage international communities and organizations to play a vital role in supporting Syrian healthcare by providing medical supplies, funding, and expertise to rebuild the healthcare infrastructure and improve the outcomes of AF and CHF, which are underdiagnosed in our region.^27^

## Conclusion

More than one in 10 patients were readmitted with new CHF after index discharge for AF from this Syrian center. Older age, VHD, DM, and hypertension were independently associated with readmission with incident CHF. DM and LVEF <40% independently increased inpatient mortality with CHF. These results stress the importance of integrated management of lifestyle risk factors and common comorbidities in AF patients to optimize outcomes in resource-depleted communities.

**Table 3.** Cox regression showing univariate and multivariable-adjusted predictors of inpatient mortality in patients readmitted with heart failure within 120 days after index discharge for atrial fibrillation hospitalization.

Inpatient mortality	Univariable analysis		Multivariable- adjusted	
Presenting characteristic	HR (95% CI)	*p*-value	HR (95% CI)	*p*-value
LVEF (<40% versus ≥40%)	5.5 (1.26-24)	0.041	6.7 (2.4-31)	0.01
Diabetes mellitus (yes versus no)	6.5 (1.3-34)	0.01	7.2 (1.9-33)	0.004
Female versus male	1.1 (0.21-5.3)	0.89	1.2 (0.6-4.7)	0.92
Age of or above 60 years versus age below 60 years	1.8 (0.71-20.6)	0.12	2.1 (0.44-5.4)	0.34
Hypertension (yes versus no)	1.24 (0.24-3.35)	0.98		
Valvular heart disease (yes versus no)	3.7 (0.56-24.2)	0.4		
Cerebrovascular disease (yes versus no)	3.6 (0.48-26.4)	0.72		
Ischemic heart disease (yes versus no)	1.1 (0.56-1.95)	0.49		
Active thyroid disease (yes versus no)	1.73 (0.19-116.2)	0.31		
Chronic kidney disease (yes versus no)	3.8 (0.1-15)	0.72		
Chronic liver disease (yes versus no)	3.7 (0.63-21.9)	0.3		
Active malignancy (yes versus no)	3 (0.05-19)	0.66		
Chronic lung disease (yes versus no)	3.6 (0.43-28.9)	0.6		
Dementia (yes versus no)	1.9 (0.2-54)	0.96		
Anaemia (yes versus no)	2.3 (0.13-18)	0.49		

CI: confidence interval. HR: hazard ratio. LVEF: left ventricular ejection fraction.

## Limitations

Data collection was limited to a single tertiary care center in Latakia. This city was relatively less affected by the Syrian conflict than the other northern and eastern regions of Syria. Therefore, our results might not be generalizable to other centers/regions, given the significant heterogeneity in the quality and level of hospital supplies and staffing. Additionally, our analysis included only routinely collected data within the medical records and by the number of patients who presented to the hospital. Therefore, other variables potentially impacting CHF admissions may have yet to be identified. As this is a single-center analysis, readmission to other centers might have been missed, and the readmission rate might have been underestimated. The study did not address treatments given during index admission, which could have affected the study’s outcomes.

## Data Availability

Data relating to this study are available upon reasonable request from the corresponding author.
